# Improving Outcomes in Cerebral Palsy with Early Intervention: New Translational Approaches

**DOI:** 10.3389/fneur.2015.00024

**Published:** 2015-02-11

**Authors:** Anna Purna Basu, Gavin Clowry

**Affiliations:** ^1^Institute of Neuroscience, Newcastle University, Newcastle upon Tyne, UK

**Keywords:** cerebral palsy, early intervention, translational medical research, diagnosis, outcome prediction

Cerebral palsy (CP) is defined as a group of permanent disorders of the development of movement and posture, causing activity limitation, attributed to non-progressive disturbances occurring in the developing fetal or infant brain (1). Lesions to the sensorimotor cortex, subcortical axon tracts, and subplate are often implicated, with other motor and non-motor areas frequently also affected. The etiology is complex and often multifactorial (2); causes include hypoxia (3), stroke (4), infection (5), trauma, and genetic factors (6). By the end of the second trimester, corticospinal axons have invaded the spinal gray matter and thalamic afferents the upper layers of the neocortex (7, 8). These systems undergo activity-dependent development (9, 10). After early brain injury, descending pathways are disrupted, with abnormal development of reflex and corticospinal circuitry (11, 12). Movement abnormalities are initially subtle but develop subsequently (13, 14). Aberrant post-lesional plasticity undoubtedly contributes to CP. It is misleading to suppose that developmental mechanisms are self-reparative. The challenge is to understand activity-dependent fine-tuning of neural circuitry during *normal* development and promote desirable plasticity while limiting undesirable effects following developmental lesions. However, before proposing interventions, we have to improve our outcome prediction skills.

Cerebral palsy affects 2/1000 live births (15): its prevalence is several times greater than spinal cord injury (SCI) and amyotrophic lateral sclerosis (ALS) (16), which also affect the corticospinal system. However, a Web of Science literature search for 2010–2014 using the phrases “cerebral palsy” (excluding supranuclear palsy), “spinal cord injury,” and “amyotrophic lateral sclerosis” returned fewer publications for CP (6653) than SCI (16147) or ALS (8258). For the flagship journals Nature Neuroscience and Neuron, the difference was greater: just one return for CP compared with 39 for SCI and 63 for ALS. Thus, CP, which causes lifelong and often severe disability, is under-researched compared with other conditions that engage neuroscientists and neurologists. We proposed a “Frontiers in Neurology Research Topic” on improving outcomes in CP with early intervention, as a forum to promote CP-related research. We involved authors with expertise ranging from signaling pathways and stem cells through functional imaging and neurophysiology to non-invasive interventions in humans. Articles include long and short reviews, original research, and opinion pieces from basic scientists and clinicians. We achieved our aim in covering prediction of outcomes of pre- and perinatal lesions, basic research in animal models and human subjects, and ideas for, and trials of, early interventions.

Hadders-Algra (17) sets the scene with a comprehensive review summarizing early brain development and discussing the effect of lesions and implications for early diagnosis and intervention. Marcroft et al. (18) review developments in movement recognition technology for classifying spontaneous general movements in high-risk infants. This theme of technology-assisted assessment is further continued by Allievi et al. (19) who focus on the use of instrumented toys and robot-assisted assessment tools with functional MRI so that functional brain activity can be mapped in health and disease even in infancy. Taking a different approach to early detection, Douglas-Escobar et al. (20) explore the potential value of two serum biomarkers of brain damage and neurodevelopmental outcomes in neonates with hypoxic–ischemic encephalopathy (HIE), namely UCH-LI and GFAP.

We received a number of basic research articles relating to early brain injury. Alagappan et al. (21) show that the increase in neural precursor cell growth and proliferation in the subventricular zone after injury depends on insulin-like growth factor receptor signaling as well as EGRF. They discuss how the nature of the culture medium used could have obscured this important finding until now. Again at a signaling pathway level, Frasch (22) considers the role of adenosine monophosphate kinase (AMPK) in inducing adaptive fetal brain shut-down and suppressing pro-inflammatory responses in the context of worsening acidemia during labor. This opinion paper accompanies the article by Xu et al. (23), which explores in an ovine model the complex relationship between preceding chronic fetal hypoxia, acute and worsening acidosis, timing and duration of adaptive brain shut-down, and the degree of brain inflammation. They suggest that EEG monitoring in addition to fetal heart rate monitoring during labor may identify earlier those infants at risk of developing severe acidosis. The ovine model does shed light on the human situation but as ever, extrapolations between species must be done with caution. Clowry et al. (24) address this issue in detail in a review of the suitability of various animal models for testing early intervention approaches in CP.

Moving from physiology to histology and detailed longitudinal neuroimaging, Kostovic et al. (25) characterize white matter lesions in preterm infants in terms of the developmental dynamics of “cellular compartments in the cerebral wall,” demonstrating how if the precise location and timing of the insult is known, the axonal pathways affected can be predicted. Mackey et al. (26) also use neuroimaging to understand outcome, but in the context of established unilateral CP. In this setting, diffusion-weighted MRI-based fractional anisotropy in the posterior limb of the internal capsule correlates with upper limb functional assessments. They also demonstrate deficits in intracortical and interhemispheric inhibition in those with poor upper limb function.

We also solicited articles on early intervention approaches. Two of these covered cell therapy. Gonzales-Portillo et al. (27) explore the potential for stem cell therapy in neonatal HIE and the outstanding clinical issues to be addressed, while Li et al. (28) discuss umbilical cord blood cell therapies in preterm infants, focusing on white matter injury. The other two articles address non-invasive approaches in infants with unilateral brain damage. Friel et al. (29) review current knowledge of corticospinal tract development including genetic and activity-dependent influences, and describe interventional approaches potentially applicable to hemiplegic CP. Finally, Basu et al. (30) take a clinical standpoint, describing the problems faced in hemiplegic CP, traditional approaches to management and their limitations, and interventions currently under investigation in infants.

We thank everyone who has supported this enterprise by submitting or reviewing manuscripts. We hope this Research Topic will serve its purpose of showcasing some of the fascinating advances in CP research, and raising the profile of this important condition to promote further investigation, ultimately for the benefit of those affected.

## Conflict of Interest Statement

The authors declare that the research was conducted in the absence of any commercial or financial relationships that could be construed as a potential conflict of interest.
